# Concurrent Infections of *Giardia duodenalis*, *Enterocytozoon bieneusi*, and *Clostridium difficile* in Children during a Cryptosporidiosis Outbreak in a Pediatric Hospital in China

**DOI:** 10.1371/journal.pntd.0002437

**Published:** 2013-09-12

**Authors:** Lin Wang, Lihua Xiao, Liping Duan, Jianbin Ye, Yaqiong Guo, Meijin Guo, Lili Liu, Yaoyu Feng

**Affiliations:** 1 State Key Laboratory of Bioreactor Engineering, School of Resources and Environmental Engineering, East China University of Science and Technology, Shanghai, People's Republic of China; 2 Division of Foodborne, Waterborne, and Environmental Diseases, Centers for Disease Control and Prevention, Atlanta, Georgia, United States of America; Christian Medical College, India

## Abstract

**Background:**

Over 200 cryptosporidiosis outbreaks have been reported, but little is known if other enteric pathogens were also involved in some of these outbreaks. Recently, an outbreak of cryptosporidiosis linked to poor hygiene by two *Cryptosporidium hominis* subtypes occurred in a pediatric hospital ward (Ward A) in China, lasting for more than 14 months. In this study, the concurrence during the outbreak of three other enteric pathogens with a similar transmission route, *Giardia duodenalis*, *Enterocytozoon bieneusi*, and *Clostridium difficile*, was assessed.

**Methods/Principal Findings:**

The occurrence of *G. duodenalis*, *E. bieneusi*, and *C. difficile* in 78 inpatients from Ward A and 283 and 216 inpatients from two control wards (Wards C and D) in the same hospital was examined using molecular diagnostic tools. Significantly higher infection rates were found in children in Ward A for all study pathogens than in Wards C and D (*P*<0.01): 9.5% versus 1.4% and 0% for *G. duodenalis*, 10.8% versus 2.8% and 3.7% for *E. bieneusi*, and 60.8% versus 37.8% and 27.8% for *C. difficile*, respectively. These differences were mostly seen in children ≤12 months. Enteric pathogen-positive children in Ward A (31/58 or 53.4%) were more likely to have mixed infections than those in Ward C (4/119 or 3.4%) or D (5/68, 7.4%; *P*<0.01). Having cryptosporidiosis was a risk factor for *G. duodenalis* (OR = 4.3; *P = *0.08), *E. bieneusi* (OR = 3.1; *P = *0.04), and *C. difficile* (OR = 4.7; *P*<0.01) infection. In addition, a lower diversity of *G. duodenalis*, *E. bieneusi*, and *C. difficile* genotypes/subtypes was observed in Ward A.

**Conclusions/Significance:**

Data from this study suggest that multiple pathogens were concurrently present during the previous cryptosporidiosis outbreak. Examination of multiple enteric pathogens should be conducted when poor hygiene is the likely cause of outbreaks of diarrhea.

## Author Summary

The transmission of *Giardia duodenalis*, *Enterocytozoon bieneusi*, and *Clostridium difficile* is poorly understood in developing countries despite their wide occurrence. Because they are transmitted by the same fecal-oral route as *Cryptosporidium*, in this study, we have examined the occurrence of these enteric pathogens in children during a cryptosporidiosis outbreak in a pediatric hospital in China. Using molecular diagnostic tools, we have detected significantly higher infection rates of these enteric pathogens in the outbreak ward than in two control wards in the same hospital. We have also shown a much higher occurrence of these pathogens in children having cryptosporidiosis than those having no cryptosporidiosis. We have demonstrated that the genetic diversity of enteric pathogens is much lower in the outbreak ward than in control wards. Therefore, other enteric pathogens are concurrently present during the cryptosporidiosis outbreak, and examinations for multiple enteric pathogens should be conducted when poor hygiene is considered the likely cause of outbreaks of diarrhea.

## Introduction

*Cryptosporidium* is a significant cause of diarrhea in humans worldwide [1]. Humans can acquire *Cryptosporidium* infections through the fecal-oral route via direct person-to-person or animal-to-person contact, or ingestion of contaminated water or food [2]. Thus far, over 200 waterborne, foodborne, person-to-person, and zoonotic cryptosporidiosis outbreaks have been reported [3], [4]. However, whether other co-pathogens were involved in some of these outbreaks remains largely unexamined.

Similar to *Cryptosporidium*, pathogens like *Giardia duodenalis*, *Enterocytozoon bieneusi*, and *Clostridium difficile* are also significant causes of diarrhea in humans worldwide and can be transmitted from persons to persons by the same fecal-oral route involved in cryptosporidiosis occurrence [1], [5], [6]. All of these pathogens are major causes of healthcare-associated infections, especially *Clostridium difficile* [7]–[9]. Despite their wide occurrence, the epidemiology of these enteric pathogens is largely unclear in developing countries. Only limited data exist on the molecular epidemiology of these pathogens in China [10]–[16].

In one recent molecular epidemiologic study on *Cryptosporidium* in in-patients from three pediatric hospitals, P. R. China, we identified an extended outbreak of cryptosporidiosis in a pediatric hospital ward (Ward A, Hospital I), with more than 50% (38/74) children affected by two *C. hominis* subtypes (IaA14R4 and IdA19) during a 14-month period (Sep. 2007–Oct. 2009) [17]. The infection rate in Ward A was significantly higher than the overall rates in Hospitals I (2.8%), II (0.6%) and III (0.4%). The diversity of *Cryptosporidium* species and *C. hominis* subtypes were significantly lower in Ward A than in other wards/hospitals, with only one species (*C. hominis*) and two *C. hominis* subtypes (IaA14R4 and IdA19) being found in 38 patients in Ward A while four species of *Cryptosporidium* and six *C. hominis* subtypes being found in 62 patients in other wards and hospitals [17].

Because concurrent infections of multiple pathogens are sometimes involved in gastroenteritis in hospitalized children [18], [19], in the present study, we retrospectively compared the infection rates and subtype distribution of *G. duodenalis*, *E. bieneusi*, and *C. difficile* in hospitalized children in Ward A with those in two control wards in the same hospital: Ward C for patients having hemophilia, anemia, and neurological diseases, and Ward D for patients having general surgeries. This was the first study to use genotyping and subtyping tools to investigate the transmission of multiple enteric pathogens during a cryptosporidiosis outbreak.

## Methods

### Ethics statement

Written informed consent was obtained from the parents or guardians of the children. This study was approved by the Ethics Committee of the East China University of Science and Technology.

### Clinical specimens and study design

All specimens for this study were collected from in-hospital children during September 2007–October 2009 as described [17]. These children were hospitalized mostly due to non-gastrointestinal illness: Ward A for patients with various congenital or inherited diseases from a local welfare institute; Ward C for children attending the Department of Endocrinology, Hematology and Neurology; and Ward D for children attending the Department of General Surgery.

In this study, Ward A (*Cryptosporidium* infection rate = 51.4%), where the cryptosporidiosis outbreak occurred, was regarded as the case ward, while two other wards (Wards C and D; *Cryptosporidium* infection rates = 1.8% and 2.3%, respectively) in the same hospital (Hospital I in Shanghai, China) without cryptosporidiosis outbreak were regarded as the control wards. Overall, 573 children, including 74 from Ward A (age range: 1–192 months; mean age: 20.7 months), 283 from Ward C (age range: 1–168 month; mean age: 41.3 months), and 216 from Ward D (age range: 1–216 months; mean age: 43.8 months), were examined for the occurrence and genotype/subtype distribution of *G. duodenalis*, *E. bieneusi*, and *C. difficile*. In addition, 2,672 children from other known or unknown wards in Hospital I (age range: 0–228 months; mean age: 46.9 months), 489 children from Hospital II (age range: 0–192 months; mean age: 37.2 months age), and 311 children from Hospital III (age range: 1–159 months; mean age: 40.4 months) in the same city, were also examined for *G. duodenalis*. Information on age, gender, and the occurrence of diarrhea as defined by the attending physicians was collected for each patient as previously described [17].

### Molecular diagnosis of enteric pathogens

Genomic DNA was extracted from 0.2 ml of fecal materials using a FastDNA SPIN Kit for Soil (BIO 101, Carlsbad, CA). To detect *G. duodenalis*, a 532-bp fragment of the triosephosphate isomerase (*tpi*) gene was amplified by nested PCR [20]. A 511-bp fragment of the β-*Giardia* (*bg*) and a 530-bp fragment of the glutamate dehydrogenase (*gdh*) gene were further amplified from DNA of the *tpi*-positive specimens [21], [22]. *Giardia duodenalis* genotypes and subtypes were determined using the established nomenclature system based on multilocus sequence data [22].

A ∼392-bp fragment of the *rRNA* gene containing the entire internal transcribed spacer (*ITS*) was amplified and sequenced to detect and identify *E. bieneusi* genotypes [23]. Genotypes of *E. bieneusi* were named according to established nomenclature [23], [24]. A PCR based on the *tcdB* gene was used to detect *C. difficile* [25]. *Clostridium difficile* in tcdB-positive specimens was subtyped by sequence analysis of the *slpA* gene as previously described [9].

### Sequence analysis

All positive PCR products generated in the study were directly sequenced using Big Dye Terminator v3.1 Cycle Sequencing Kits (Applied Biosystems, Foster City, CA) and an ABI 3130 Genetic Analyzer (Applied Biosystems). Sequences were assembled using ChromasPro (version 1.5) software (http://technelysium.com.au/?page_id=27). The accuracy of the sequencing reads was confirmed by bidirectional sequencing. The nucleotide sequences of *G. duodenalis*, *E. bieneusi*, and *C. difficile* genotypes/subtypes obtained were aligned with reference sequences of each genetic locus downloaded from GenBank using ClustalX (http://www.clustal.org/). A neighbor-joining analysis of the aligned sequences was performed with the program Mega 5 (http://www.megasoftware.net/). Unique nucleotide sequences generated from the study were deposited in GenBank under accession numbers JX994231-JX994292.

### Statistical analysis

The χ^2^ test was used to compare infection rates between Ward A and the control wards. The same method was used to analyze the association between infection and age, gender, or diarrhea status. The strength of the association was measured using the odds ratio (OR). Differences were considered significant at *P*≤0.05. All statistical analyses were performed using the SPSS Statistics 17.0 (SPSS Inc, Chicago, IL).

## Results

### Infection rates of enteric pathogens in case and control wards

**Figure 1:**
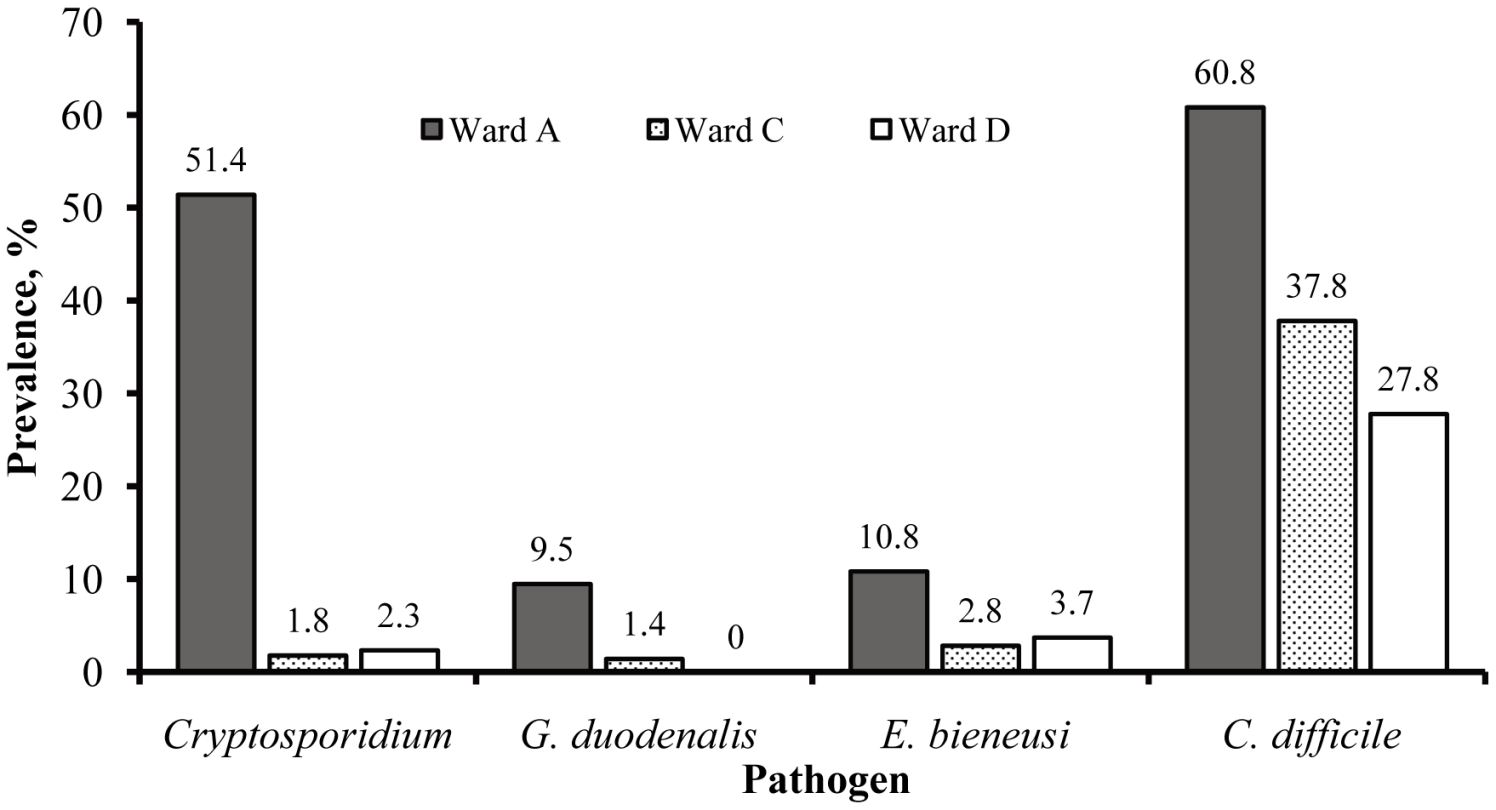
Infection rates of study pathogens in case and control wards. There were 74 children in case ward (Ward A), 283 and 216 children in control wards (Wards C and D).

Only four parasites including *Cryptosporidium*, *G. duodenalis*, *E. bieneusi* and *C. difficile* were analyzed in this study and no examinations of bacteria or viruses were conducted. The *Cryptosporidium* infection rates were 51.4% (38/74) in case Ward A while 1.8% (5/283) and 2.3% (5/216) in control Wards C and D, respectively [17]. Among the 74 specimens from the case Ward A, seven (9.5%) were positive for *G. duodenalis* at the *tpi* locus (Figure 1). In contrast, only 4 of 283 (1.4%) and 0 of 216 (0%) specimens from the control Wards C and D were positive (Figure 1). The difference in *G. duodenalis* infection rates between the case (A) and control wards (C and D) was significant (*P*<0.01; Table 1). In addition, 4 of 1,019 (0.4%) children from other wards in Hospital I, 17 of 1,653 (1.0%) children from unknown wards in Hospital I, 3 of 489 (0.6%) children from Hospital II, and 5 of 311 (1.6%) children from Hospital III were also positive for *G. duodenalis*. The prevalence of giardiasis in children from these locations was significantly lower than the prevalence in Ward A (*P*<0.01).

**Table 1:** Distribution of *Giardia duodenalis*, *Enterocytozoon bieneusi*, and *Clostridium difficile* infections in pediatric inpatients by ward and *Cryptosporidium* infection status.

		*G. duodenalis*			*E. bieneusi*			*C. difficile*		
Factors	Specimen size	Positive no. (%)	OR (95% CI^a^)	*P* b	Positive no. (%)	OR (95% CI^a^)	*P* b	Positive no. (%)	OR (95% CI^a^)	*P* b
Hospital ward										
Case Ward (A)	74	7 (9.5)	12.9 (3.7, 45.3)	**<0.01**	8 (10.8)	3.7 (1.5, 8.9)	**0.01**	45 (60.8)	3.1 (1.9, 5.1)	**<0.01**
Control Wards (C and D)	499	4 (0.8)	Reference		16 (3.2)	Reference		167 (33.5)	Reference	
Cryptosporidiosis status										
Yes	48	3 (6.3)	4.3 (1.1, 16.8)	0.08	5 (10.4)	3.1 (1.1, 8.7)	**0.04**	34 (70.8)	4.7 (2.5, 9.1)	**<0.01**
No	525	8 (1.5)	Reference		19 (3.6)	Reference		178 (33.9)	Reference	

a CI: confidence interval.

b Bold numbers: *P* values≤0.05 by Chi-square test.

Among the 573 specimens examined, 24 (4.2%) were positive for *E. bieneusi* at the *ITS* locus, with eight positives in each ward. The infection rate of *E. bieneusi* in Ward A (10.8%) was significantly higher than those in Ward C (2.8%) and D (3.7%) (*P* = 0.01; Figure 1; Table 1). Altogether, 212 of the 573 specimens were positive for *C. difficile* at the *tcdB* locus, with 45/74 (60.8%), 107/283 (37.8%), and 60/216 (27.8%) in Wards A, C, and D being positive, respectively (Figure 1). The infection rate of *C. difficile* was significantly higher in Ward A than in Wards C and D (*P*<0.01; Table 1).

### Concurrent infections with multiple pathogens in case and control wards

Concurrent infections of multiple pathogens, including *Cryptosporidium*, *G. duodenalis*, *E. bieneusi*, and *C. difficile*, were detected in both the case and control wards. Comparing with the control Wards C and D, Ward A had a significantly higher overall infection rate of enteric pathogens (58/74 or 78.4% versus 119/283 or 42.0% and 68/216 or 31.5%; *P*<0.01). Over half of children with enteric pathogens in Ward A (31/58 or 53.4%) were concurrently infected with two or more pathogens, while only a small number of children with enteric pathogens in Ward C (4/119 or 3.4%) or D (5/68, 7.4%) were infected with multiple pathogens (*P*<0.01).

In this study, children who had cryptosporidiosis during the outbreak were more likely to be infected with other enteric pathogens (Table 1). Among the 573 children examined for all four pathogens, 48 were previously diagnosed as having cryptosporidiosis. These *Cryptosporidium*-positive children had higher infection rates of *G. duodenalis* (6.3% versus 1.5%; *P* = 0.08), *E. bieneusi* (10.4% versus 3.6%; *P = *0.04), and *C. difficile* (70.8% versus 33.9%; *P*<0.01) than *Cryptosporidium*-negative children (Table 1).

### Occurrence of enteric pathogens by age and gender

The age distribution of *G. duodenalis*, *E. bieneusi*, and *C. difficile* infections in 573 children from Wards A, C, and D is shown in Table 2. Infection rates of *G. duodenalis* were similar among all age groups (*P*>0.05). In contrast, children ≤6 months were more likely infected with *E. bieneusi* (11/99 or 11.1% versus 12/473 or 2.5% for other age groups, *P*<0.01), and children ≤12 months were more likely infected with *C. difficile* (124/277 or 44.8% versus 88/295 or 29.8% for other age groups, *P*<0.01; Table 2). Among children under 12 months, infection rates of all three study pathogens were significantly higher in Ward A than in control wards (5/59 or 8.5% versus 1/218 or 0.5%, *P*<0.01 for *G. duodenalis*; 7/59 or 11.9% versus 8/218 or 3.7%, *P* = 0.03 for *E. bieneusi*; 38/59 or 64.4% versus 86/218 or 39.4%, *P*<0.01 for *C. difficile*). In contrast, in children older than 12 months, only *G. duodenalis* was significantly more prevalent in Ward A than in the controls (2/15 or 13.3% versus 3/280 or 1.1%, *P* = 0.01 for *G. duodenalis*; 1/15 or 6.7% versus 7/280 or 2.5%, *P* = 0.88 for *E. bieneusi*; 7/15 or 46.7% versus 81/280 or 28.9%, *P* = 0.14 for *C. difficile*; Table 2). No gender difference was seen in the occurrence of *G. duodenalis*, *E. bieneusi*, and *C. difficile* infections in this study (*P*>0.05; Table 2).

**Table 2:** Distribution of study pathogen infections in case (A) and control (C and D) wards by age, gender, and diarrhea status.

Group^a^	Positive/total no. (%) for *G. duodenalis*			Positive/total no. (%) for *E. bieneusi*			Positive/total no. (%) for *C. difficile*		
	Total	Ward A	Wards C & D	Total	Ward A	Wards C & D	Total	Ward A	Wards C & D
Age (months)									
1–6	2/99 (2.0)	1/39 (2.6)	1/60 (1.7)	11/99 (11.1)	7/39 (17.9)	4/60 (6.7)	38/99 (38.4)	26/39 (66.7)	12/60 (20.0)
7–12	4/178 (2.2)	4/20 (20.0)	0/158 (0)	4/178 (2.2)	0/20 (0)	4/158 (2.5)	86/178 (48.3)	12/20 (60.0)	74/158 (46.8)
>12	5/295 (1.7)	2/15 (13.3)	3/280 (1.1)	8/295 (2.7)	1/15 (6.7)	7/280 (2.5)	88/295 (29.8)	7/15 (46.7)	81/280 (28.9)
Gender									
Male	6/388 (1.5)	4/43 (9.3)	2/345 (0.6)	13/388 (3.4)	2/43 (4.7)	11/345 (3.2)	137/388 (35.3)	27/43 (62.8)	110/345 (31.9)
Female	5/184 (2.7)	3/31 (9.7)	2/153 (1.3)	10/184 (5.4)	6/31 (19.4)	4/153 (2.6)	75/184 (40.8)	18/31 (58.1)	57/153 (37.3)
Diarrhea									
Yes	5/223 (2.2)	3/43 (7.0)	2/180 (1.1)	11/223 (4.9)	3/43 (7.0)	8/180 (4.4)	73/223 (32.7)	27/43 (62.8)	46/180 (25.6)
No	6/350 (1.7)	4/31 (12.9)	2/319 (0.6)	13/350 (3.7)	5/31 (16.1)	8/319 (2.5)	139/350 (39.7)	18/31 (58.1)	121/319 (37.9)

a One child from Ward C and one child from Ward D did not have age and gender information, respectively.

### Distributions of *G. duodenalis*, *E. bieneusi*, and *C. difficile* genotypes/subtypes

**Figure 2:**
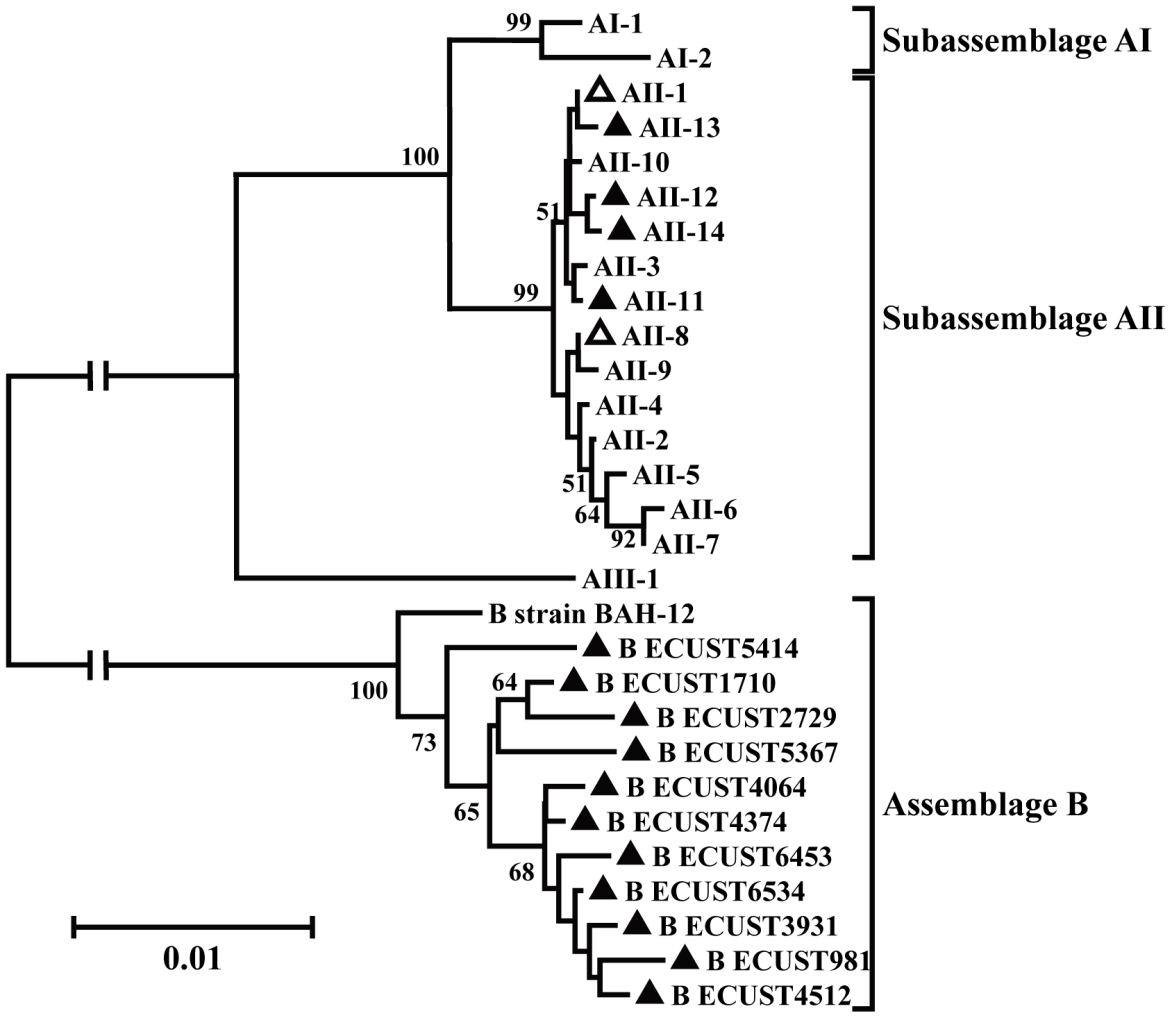
Phylogenetic relationship of multilocus sequence subtypes of *Giardia duodenalis*. Sequences from this and a previous study [22] are included in the analysis. The relationship of multilocus sequence subtypes was inferred by a neighbor-joining analysis of concatenated sequences of the *gdh*, *tpi*, and *bg* fragments, based on the p-distance model. Bootstrap values >50% are shown. Novel and known genotypes identified in this study are indicated by black and white triangles, respectively.

The distribution of *G. duodenalis* multilocus subtypes was different between case and control wards. In Ward A, six of the seven specimens positive for *G. duodenalis* at the *tpi* locus were also positive at the *bg* and *gdh* loci, and all of them belonged to the multilocus subtype AII (Table 3; Figure 2). In contrast, both multilocus subtype AII and subtypes belonging to the assemblage B (2 cases each) were found in Ward C. Similarly, both AII and B were detected in other known or unknown wards in Hospital I, and in Hospitals II and III (Table 3).

**Table 3:** Distribution of *Giardia duodenalis*, *Enterocytozoon bieneusi*, and *Clostridium difficile* genotypes or subtypes.

Pathogen	Source	No. positive/sample size (%)^a^		Genotypes/subtypes (No. positive)^b^
			Dominant	Others
*G. duodenalis*	Ward A, Hospital I	7/74 (9.5)	AII (6)	
	Ward C, Hospital I	4/283 (1.4)		AII (2), B (2)
	Ward D, Hospital I	0/216 (0)		
	Other wards, Hospital I	4/1019 (0.4)		AII (1), B (3)
	Unknown wards, Hospital I	17/1653 (1.0)	AII (12)	B (3)
	Hospitals II and III	8/800 (1.0)		AII (4), B (3)
*E. bieneusi*	Ward A, Hospital I	8/74 (10.8)	Peru 11 (4)	SH1–4 (1 each)
	Ward C, Hospital I	8/283 (2.8)		Peru 11, EbpC, EbpA, SH2, SH5–8 (1 each)
	Ward D, Hospital I	8/216 (3.7)		Peru 11, EbpA, D, SH2, SH9–12 (1 each)
*C. difficile*	Ward A, Hospital I	45/74 (60.8)	fr-01 (15)	og39-01 (6), kr-03 (5), fr-sh1 (2), fr-sh4 (1), sh-01 (1), hr-01 (1), gc11-01 (1), xr-03 (1), kr-03 & sh-01 (4), kr-03 & og39-01 (1), sh-01 & og39-01 (2)
	Ward C, Hospital I	107/283 (37.8)	kr-03 (18)	og39-01 (13), fr-01 (13), hr-01 (7), gr-01 (5), sh-01 (3), xr-03 (2), fr-sh3 (1), gc11-01 (1), og39-sh1 (1), smz-02 (1), j52-01 (1), yok-01 (1), serogroup D (1), kr-03 & og39-01 (3), gc11-01 & xr-sh1 (1), kr-03 & sh-01 (1), kr-03 & serogroup D (1)
	Ward D, Hospital I	60/216 (27.8)	kr-03 (13)	og39-01 (9), hr-01 (4), gr-01 (4), fr-01 (3), fr-sh1 (1), fr-sh2 (1), fr-sh4 (1), og39-sh1 (1), sh-02 (1), yok-01 (1), nc0930-01 (1), kr-03 & sh-01 (3), kr-03 & xr-03 (1), kr-03 & og39-01 (1), sh-01 & og39-01 (1)

a Four *G. duodenalis*-positive specimens belonging to *tpi* A2, including one from Ward A, two from unknown wards, and one from Hospital III, failed in multilocus subtyping; 52 *C. difficile*-positive specimens, including 5 from Ward A, 33 from Ward C, and 14 from Ward D, failed in *slpA* subtyping.

b For *G. duodenalis*, 6 and 11 multilocus sequence types belonging to AII and B were found, respectively; *G. duodenalis* AII-11 to AII-14, *E. bieneusi* SH1–12, and *C. difficile* fr-sh1–4, og39-sh1, sh-01, sh-02, xr-sh1 are new genotypes or subtypes found in this study.

**Figure 3:**
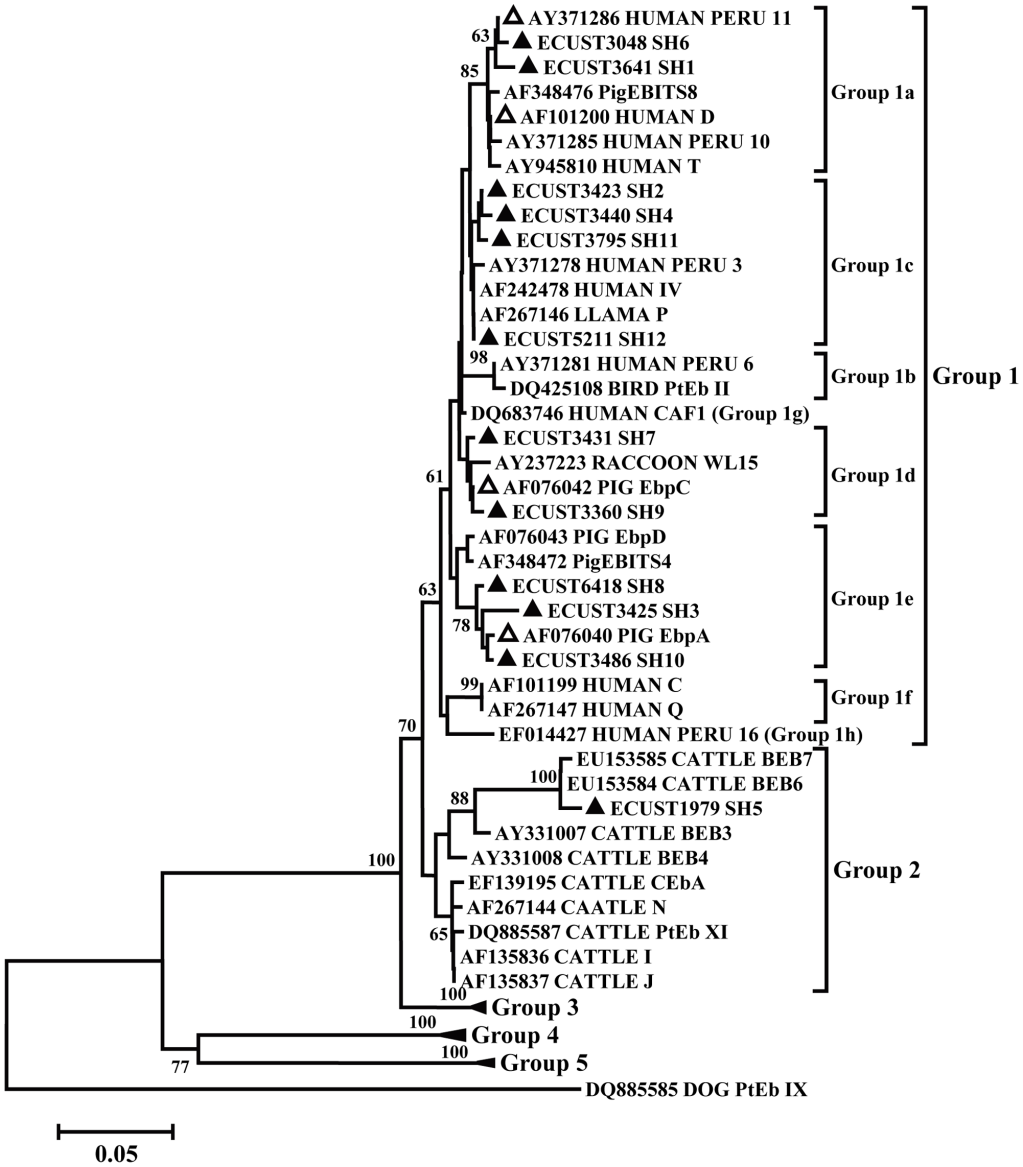
Phylogenetic relationship of *Enterocytozoon bieneusi* genotypes. The relationship of genotypes identified in this study and some other genotypes in GenBank was inferred by a neighbor-joining analysis of *ITS* sequences, based on the p-distance model. Bootstrap values >50% are shown. Novel and known genotypes identified in this study are indicated by black and white triangles, respectively.

Four known genotypes of *E. bieneusi* were found in this study, with Peru 11 as the dominant one (in 6 cases). The other three includes EbpC (1 case), EbpA (2 cases), and D (1 case). Twelve novel genotypes (SH1–12) were found in this study, with SH2 in three cases and all other genotypes in one case each (Table 3). All 16 *E. bieneusi* genotypes except SH5 belonged to Group 1 phylogenetically, while genotype SH5 belonged to Group 2 (Figure 3). Higher occurrence of *E. bieneusi* genotype Peru 11 was seen in Ward A (4/8 genotyped) than in Wards C and D (1/8 genotyped each; Table 3).

**Figure 4:**
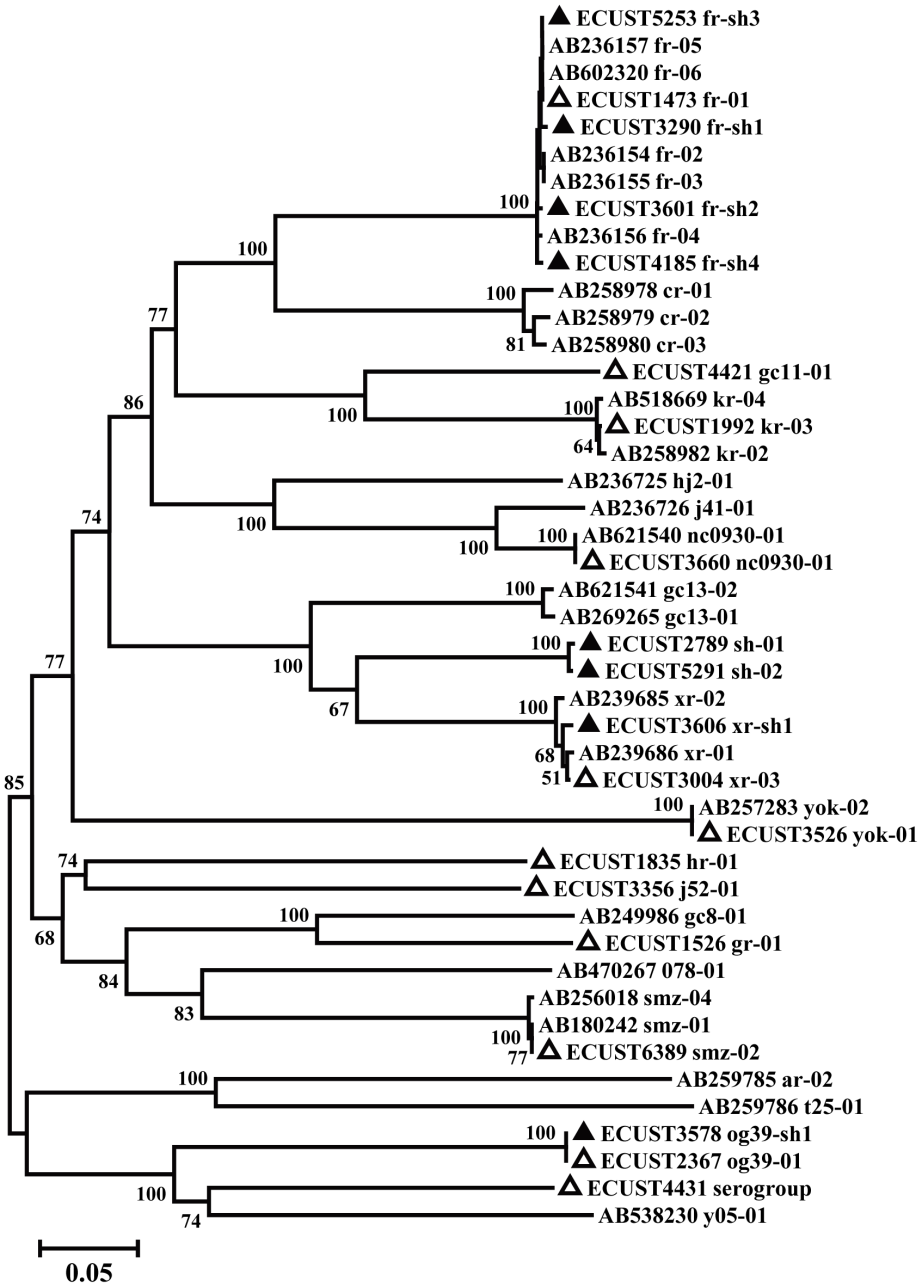
Phylogenetic relationship of *Clostridium difficile* subtypes. The relationship of subtypes identified in this study and other subtypes in a previous study [9] was inferred by a neighbor-joining analysis of *slpA* sequences, based on the p-distance model. Bootstrap values >50% are shown. Novel and known subtypes identified in this study are indicated by black and white triangles, respectively.

Among the 212 *C. difficile*-positive specimens based on PCR analysis of the *tcdB* gene, 160 specimens were subtyped at the *slpA* locus successfully. In total, 20 *slpA* subtypes were obtained, including 8 novel ones (Table 3). Most of the novel subtypes were genetically close to subtypes previously reported, although two of them, sh-01 and sh-02, had very different sequences and formed an independent clade in the phylogenetic tree (Figure 4). The most common subtype in Ward A was fr-01 (15/40 *slpA-*positive cases), compared to kr-03 in control Wards C (23/74 *slp-A* positive cases) and D (18/46 *slp-A* positive cases; Table 3).

### Pathogen occurrence and diarrhea

A significantly higher diarrhea rate was observed in Ward A than in control Wards C and D (43/74 or 58.1% versus 180/499 or 36.1%, *P*<0.01). Infection with *Cryptosporidium* was significantly associated with the occurrence of diarrhea (OR = 1.95, p = 0.002). However, a large number of asymptomatic *G. duodenalis*, *E. bieneusi*, and *C. difficile* infections were observed in both case and control wards in this study(Table 2). None of the three pathogens were significantly associated with the occurrence of diarrhea in the pediatric inpatients (*P*>0.05; Table 2). None of the dominant genotypes/subtypes of the three study pathogens were significantly associated with the occurrence of diarrhea (*P*>0.05; data not shown). In addition, in Ward A, the difference in diarrhea rates between children with multiple infections and children with single infection was not significant (17/31 or 54.8% versus 17/27 or 63.0%, *P = *0.51). This was also the case in the control Wards C and D (3/9 or 33.3% versus 56/178 or 31.5%, *P* = 1.0).

## Discussion

Molecular epidemiological investigations have improved our understanding of the transmission of enteric pathogens, including those examined in the present study [2], [7]–[9], [14]. They are especially useful in identifying the occurrence of outbreaks, linking seemingly un-associated cases, and tracking infection sources. In the present study, using genotype and subtype tools, we retrospectively identified concurrent transmission of *G. duodenalis*, *E. bieneusi*, and *C. difficile* during a cryptosporidiosis outbreak previously identified in Ward A of Hospital I in Shanghai, China. This is reflected by higher infection rates and lower genetic diversity of these enteric pathogens in Ward A than in control wards.

The low infection rates of *G. duodenalis* in wards other than Ward A in Hospital I (0–1.4%), Hospitals II (0.6%), and III (1.6%) are similar to those reported in out-patients and inpatients (0.2–0.6%) and the general population (2.5%) in China [11], [26]–[29]. Likewise, infection rates of *E. bieneusi* in hospitalized children in Wards C (2.8%) and D (3.7%) are also very low. In contrast, significantly higher infection rates of *G. duodenalis* (9.5%; *P*<0.01) and *E. bieneusi* (10.8%; *P* = 0.01) were seen in Ward A, indicating that these pathogens were transmitted frequently within this ward during the cryptosporidiosis outbreak. Although a high carriage of *C. difficile* was found in control Wards C (37.8%) and D (27.8%), this is similar to the 16–35% carriage of *C. difficile* in hospital inpatients in other countries [30], and significantly lower than the infection rate of *C. difficile* in Ward A (60.8%; *P*<0.01). Previously, no data existed on the prevalence of *C. difficile* in hospitalized children in China, although an infection rate of 9.5% was reported in adults in Shanghai [14].

The low genetic diversity of *G. duodenalis*, *E. bieneusi*, and *C. difficile* found in Ward A versus in control wards also supports the hypothesis of concurrent transmission of these enteric pathogens during the cryptosporidiosis outbreak. For *G. duodenalis*, AII was the only subtype seen in Ward A, although assemblage B and both AI and AII subtypes of assemblage A are commonly found in humans around the world (7), and they were all found in other wards and hospitals in the present study (Table 3). For *E. bieneusi*, Peru 11 was the dominant genotype in Ward A, being found in half of the genotyped specimens, while it was only found in one of eight specimens each genotyped in Wards C and D (Table 3). For *C. difficile*, the fr-01 subtype was dominant in Ward A and accounted for 1/3 of all *C. difficile* infections in this ward, whereas the most prevalent genotype kr-03 in control Wards C and D accounted for less than 1/5 of all *C. difficile* infections (Table 3).

Outbreaks involving multiple enteric pathogens have been infrequently reported and, in the investigations of the few such outbreaks, sewage contamination of water or food was often the main cause for concurrent transmission of multiple enteric pathogens. For example, a waterborne outbreak of *Shigella sonnei*, *Giardia*, and *Cryptosporidium* infections on a Lake Michigan dinner cruise was caused by contamination of potable water with diluted sewage as the result of storm runoff in the cruise ship [31]. Another waterborne outbreak of gastroenteritis with multiple etiologies in resort island visitors and residents in Ohio in 2004 was caused by sewage contamination of groundwater [32]. Likewise, a national multi-pathogen outbreak of diarrheal illness in Botswana in 2006 was caused by sewage contamination of the environment during heavy rains in late 2005 and early 2006 [33]. Similarly, contact with manure from calves was responsible for two multi-pathogen outbreaks at a farm day camp in Minnesota [34]. In the present study, over half of the patients with enteric pathogens (31/58) in Ward A were infected with more than one pathogen, compared to a very limited number of cases (9/187) in control wards (*P*<0.01). Of note is the significant association of the three enteric pathogens examined in this study and occurrences of cryptosporidiosis in these children (Table 1).

As suggested in our previous investigation of cryptosporidiosis in these children [17], poor diaper changing and hand washing practices by caregivers were probably responsible for this multi-pathogen outbreak among pediatric inpatients in Ward A, Hospital I. Children in Ward A were orphans from a welfare institute. They were taken care of by hired caregivers. In contrast, children in other wards were primarily from the general community and cared for by their parents [17]. Considering the fact that most infections in Ward A occurred in children younger than 12 months (Table 2), who mostly stayed in cribs and beds, hired caregivers in Ward A might have acted as vehicles for the disease transmission among pediatric inpatients. This is also supported by the finding that in children under 12 months, Ward A had significantly higher infection rates of all study pathogens than Wards C and D, but in children older than 12 month, Ward A had only significantly higher infection rates of *G. duodenalis* than Wards C and D.

Very few studies have been conducted on molecular epidemiology of *G. duodenalis*, *E. bieneusi*, and *C. difficile* in China [10]–[16]. The occurrence of both assemblages A and B of *G. duodenalis* in non-outbreak children is in accordance with previous findings of near equal distribution of the two genotypes in 18 *Giardia*-positive humans in Henan [11] and 8 in Anhui [10]. In contrast, the dominance of Group 1 *E. bieneusi* genotypes in children in this study is different from the dominance of Group 2 genotypes in children in Jilin [13], although we also detected a novel Group 2 genotype in a child from Ward C (Figure 3; Table 3). The high diversity of known and novel *E. bieneusi* genotypes reported in this study and previous studies [12], [13] suggests that there is a need for more studies to examine the characteristics of *E. bieneusi* transmission in humans in China.

Ribotypes 027 and 078 are recognized as leading causes of nosocomial outbreaks of *C. difficile* infection in the world [9], [15]. However, neither has been reported in China thus far [14]–[16]. Interestingly, the dominant *C. difficile slpA* subtypes fr-01 in Ward A was previously characterized as toxin A-negative and toxin B-positive (A−B+), whereas the dominant subtype kr-03 in control wards was toxin A-positive and toxin B-positive (A+B+) [9]. In a previous study, A−B+ strains were the dominant ones (24.0%) in patients in three hospitals in Beijing, Shandong and Guangzhou in China [16]. The high prevalence of A−B+ strains in China indicates that toxin B, rather than toxin A, is probably a key virulence determinant as previously suggested [35]. Nevertheless, in the present study, no significant association was found between any of the *C. difficile* subtypes and the occurrence of diarrhea, although we previously showed a link between cryptosporidiosis and diarrhea in these children [17]. A new group of *slpA* subtypes including sh-01 and sh-02 were found in many children from all three wards (Figure 3; Table 3). Further studies are needed to better understand the public health importance of this new group of subtypes.

The results of this study and our previous study [17] showed that although *Cryptosporidium* infection was associated with the occurrence of diarrhea, single-pathogen infection with *G. duodenalis*, *E. bieneusi*, or *C. difficile* was not. None of the dominant genotypes/subtypes of *G. duodenalis*, *E. bieneusi*, and *C. difficile* were significantly associated with the occurrence of diarrhea, and concurrent infections of multiple pathogens were not more associated with occurrence of diarrhea than infections with single pathogens. The lack of differences in occurrence of diarrhea between children with single-pathogen infection and children with mixed infections in this study was probably attributable to the already high diarrhea rates in Ward A (58.1%) and low occurrence of mixed infections in control Wards C and D (9 cases of mixed infections versus 178 cases of single-pathogen infection). In addition, with the exception of *Cryptosporidium*, none of the other pathogens examined in this study were among the recently identified major pathogens for moderate-to-severe diarrhea in the Global Enteric Multicenter Study [1]. This has probably also made it difficult to use attributable fraction calculation in estimating the role of mixed infections in the occurrence of diarrhea in a hospital study setting with high occurrence of diarrhea.

In conclusion, using genotyping and subtyping tools we retrospectively identified a multi-pathogen outbreak in a pediatric hospital ward. As reported previously [17], this outbreak lasted ≥14 months, with ∼60 inpatient children affected by cryptosporidiosis. Most of the *Cryptosporidium*-positive children were co-infected with *G. duodenalis*, *E. bieneusi*, or *C. difficile*. The young age of affected children and concurrent infections with multiple enteric pathogens clearly implicated poor diaper changing and hand washing by hired caregivers as the cause of the outbreak. Thus, better training of caregivers on hygienic practices such as hand washing and proper use of disposable gloves and disinfectants is needed to reduce the risk of pathogen transmission in healthcare facilities. Results of this study also highlight the importance of molecular epidemiologic investigations in understanding the transmission of enteric pathogens in hospitals.
